# Experiences of receiving and delivering a novel complex intervention for type 1 diabetes and disordered eating: Process evaluation of the Safe management of people with Type 1 diabetes and EAting Disorders studY (STEADY)

**DOI:** 10.1111/dme.70377

**Published:** 2026-06-11

**Authors:** Natalie Zaremba, Amy Harrison, Jennie Brown, Divina Pillay, Emmanouela Konstantara, Janet Treasure, David Hopkins, Judith Prins, Bastiaan de Galan, Khalida Ismail, Giesje Nefs, Marietta Stadler

**Affiliations:** ^1^ Department of Diabetes, Faculty of Life Sciences and Medicine King's College London London UK; ^2^ Florence Nightingale Faculty of Nursing, Midwifery & Palliative Care King's College London London UK; ^3^ Department of Medical Psychology Radboud University Medical Center Nijmegen the Netherlands; ^4^ Department of Psychological Medicine King's College London London UK; ^5^ Department of Psychology and Human Development Institute of Psychiatry, University College London London UK; ^6^ Diabetes Centre King's College Hospital London UK; ^7^ St Vincent Square Eating Disorder Unit Chelsea and Westminster NHS Foundation Trust London UK; ^8^ Institute of Psychiatry, Psychology and Neuroscience King's College London London UK; ^9^ Institute of Diabetes Endocrinology and Obesity King's Health Partners London UK; ^10^ Department of Internal Medicine Radboud University Medical Center Nijmegen the Netherlands; ^11^ Department of Internal Medicine Maastricht University Medical Center+ Maastricht the Netherlands; ^12^ Diabeter, Center for Focused Diabetes Care and Research Rotterdam the Netherlands; ^13^ Department of Medical and Clinical Psychology, CoRPS—Center of Research on Psychological Disorders and Somatic Diseases Tilburg University Tilburg the Netherlands; ^14^ Diabeter Centrum Amsterdam Amsterdam the Netherlands; ^15^ Department of Psychological Medicine, Diabetes, Psychology and Psychiatry Research Group King's College London London UK

**Keywords:** cognitive behaviour therapy, eating disorder, implementation science, intervention, mixed‐methods research, process evaluation, T1DE, type 1 diabetes, type 1 diabetes disordered eating

## Abstract

**Aim:**

The Safe management of people with Type 1 diabetes and EAting Disorders studY (STEADY) intervention is a novel cognitive behaviour therapy (CBT) intervention for individuals with type 1 diabetes and disordered eating (T1DE). The aim of this mixed‐method process evaluation is to assess the acceptability, appropriateness and feasibility of STEADY, and explore experiences of both participants and healthcare professionals during therapy delivery.

**Methods:**

Participants who received STEADY (*n* = 13) and healthcare professionals delivering STEADY therapy (*n* = 3) completed person‐reported outcome measures (PROMs) to assess the acceptability (AIM), appropriateness (IAM) and feasibility (FIM) of STEADY and took part in semi‐structured interviews to explore experiences, therapeutic processes and implementation considerations. Interviews were analysed using thematic analysis.

**Results:**

PROM scores indicated high ratings across participants (AIM = 18.36 ± 2.01; IAM = 18.18 ± 2.64; FIM = 18.00 ± 2.32) and healthcare professionals (AIM = 17.67 ± 2.08; IAM = 17.33 ± 2.31; FIM = 17.67 ± 2.08), suggesting high likelihood of implementation success. Thematic analysis showed significant overlap in participant and therapist experiences. Key themes included integrated T1DE‐specific CBT delivery, specialist knowledge of T1DE and a strong therapeutic alliance. Both groups emphasised the need for tailored delivery, therapist expertise in T1DE and structural adaptations to standard CBT models. Participant's experiences also included a clear path of therapeutic growth and expectations of therapy. Therapists' experiences included considerations for clinical safeguarding and organisational factors impacting wider implementation.

**Conclusions:**

Our findings demonstrate that STEADY is acceptable, appropriate and feasible for individuals with mild to moderate severity T1DE. The substantial alignment between participant and therapist perspectives offers clear directions for refining the intervention and support implementation in a full‐scale RCT.

## Graphical abstract

Overview of participant and healthcare professional experiences in the STEADY process evaluation. Key themes highlight the importance of an integrated and multidisciplinary approach to type 1 diabetes and disordered eating, positive therapeutic relationship, and flexible person‐centred care.

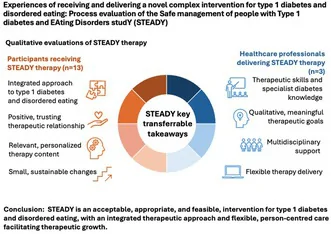

**What's new?**

**What is already known?**

- STEADY is a novel cognitive behaviour therapy‐based intervention for type 1 diabetes and disordered eating that has recently been tested in a feasibility randomised controlled trial. Process evaluations are recommended to assess complex interventions within the Medical Research Council Framework.

**What this study has found?**

- Participants and healthcare professionals in the feasibility RCT emphasized the therapeutic relationship and the integration of type 1 diabetes and disordered eating specialities within one therapeutic setting. Personalised therapy content and adaptations from standardised CBT are important structural factors for effective therapy delivery.

**What are the implications of the study?**

- This study defines the context, mechanisms and implementation of the STEADY therapy, providing a basis for further refinement and wide‐scale implementation.

## 1 INTRODUCTION

Type 1 diabetes (T1D) and disordered eating (T1DE) is a common and serious problem, affecting diabetes management outcomes, acute and long‐term complications and psychosocial outcomes in people with diabetes.^1^, ^2^ Established interventions for eating disorders are not compatible with diabetes self‐management behaviours, creating challenges for people with T1DE seeking treatment and healthcare professionals (HCPs) delivering care.^3^, ^4^ The Safe management of people with T1D and EAting Disorders studY (STEADY) is a novel complex intervention to treat T1D and T1DE. Following the development of theoretical models developed from qualitative interviews and blog analyses,^5^, ^6^ as well as an understanding of challenges faced by HCPs treating people with T1DE,^3^ we used Experience‐Based Co‐Design, a participatory action design process bringing together people with lived experience and healthcare professionals to develop STEADY using a cognitive behaviour therapy (CBT) framework integrating diabetes education.^7^, ^8^ The resulting STEADY ‘toolkit’ is an adaptable and personalised intervention that suits a wide range of T1DE presentations. The STEADY intervention was tested in a feasibility randomised controlled trial (RCT), with the intervention group receiving 12 sessions of the newly developed T1DE‐specific CBT. The feasibility RCT showed proof of concept for feasibility with high intervention completion rates, as well as improvements in self‐reported mental health scores.^9^, ^10^

STEADY was developed using the Medical Research Council (MRC) Complex Intervention Framework, which highlights the importance of the process evaluation.^7^, ^11^, ^12^ Process evaluations allow researchers and regulators to understand how the context and mechanisms of action influence outcomes and implementation of an intervention.^12^, ^13^ This can optimise evaluation and refinement of the intervention at the feasibility and piloting stage.^11^, ^13^ Within this context, the present mixed‐methods study of participant and therapist experiences explores these factors and complements the recently published quantitative evaluation of therapy delivery and audit of therapeutic tools.^14^ This will provide a greater understanding of the factors influencing the engagement, delivery and outcomes of the STEADY feasibility RCT, identify barriers and facilitators to wider implementation and inform future refinement of STEADY.

The aim of the mixed‐methods STEADY process evaluation was twofold: first, to quantitatively evaluate the feasibility, acceptability and appropriateness of STEADY, and second, to understand the experiences of receiving and delivering therapy through semi‐structured interviews with participants in the STEADY trial and HCPs delivering therapy.

## 2 METHODS

This study received ethical approval from the East of England‐Essex research ethics committee (21/EE/0235). All participants provided written informed consent.

### 2.1 Eligibility

All participants in the STEADY intervention arm were invited to take part in the process evaluation (up to *N* = 20). HCPs involved in the delivery and supervision of therapy and psychological assessment of participants were invited to take part in the process evaluation (*N* = 3; one diabetes specialist nurse [DSN] delivering therapy, one clinical psychologist delivering and supervising therapy and conducting psychological assessments, one psychiatrist supervising therapy and conducting psychiatric assessments). The flow of participants through the main STEADY RCT and process evaluation sub‐study is presented in Figure 1. Individuals eligible for the STEADY trial but who declined to take part were eligible for the process evaluation (up to *N* = 5)^9^; however, no individuals in this category consented to the sub‐study.

**FIGURE 1:**
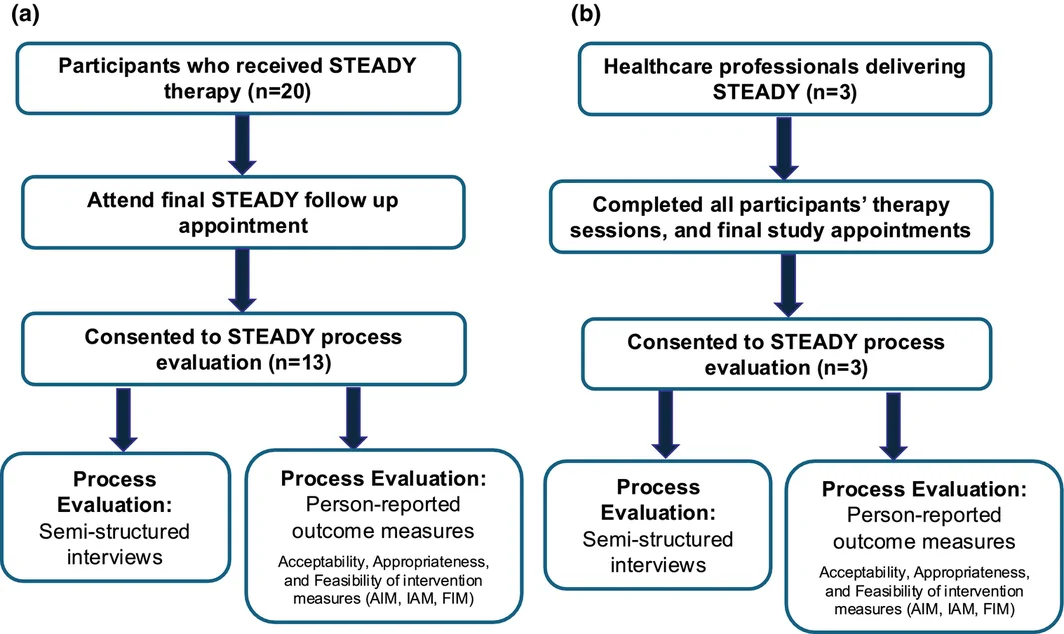
Participant flow diagram of people receiving STEADY therapy (a) and healthcare professionals delivering therapy (b).

### 2.2 Person‐reported outcome measures (PROMs) and quantitative analysis

Three validated PROMs were used to quantitatively measure the acceptability, appropriateness and feasibility of STEADY therapy: the Acceptability of Intervention Measure (AIM), Intervention Appropriateness Measure (IAM) and Feasibility of Intervention Measure (FIM).^15^ Mean scores and standard deviation were calculated. Possible scores for each PROM range from 4 to 20, with scores above 16 indicating a high likelihood of implementation success in each domain.^15^

### 2.3 Semi‐structured interviews and qualitative analysis

Semi‐structured interview guides were developed iteratively to focus on the perception of the STEADY therapy, experience of receiving therapy, experience of delivering therapy and practical and structural changes for future iterations of STEADY. The interview schedule for participants who received STEADY included questions on topics of: their initial perceptions of the STEADY intervention, engaging with the therapy content, experiences using the STEADY tools, using STEADY in daily life and the STEADY app.

The interview schedule for HCPs delivering STEADY included questions on the topics of: overall perceptions of the STEADY intervention, delivery of therapy, content and structure of therapy and tools, the STEADY app and clinician platform and future of STEADY and wider implementation.

Interviews were conducted by NZ in‐person or via videocall after the final study visit in the main STEADY trial (STEADY intervention group), and after all participants had completed the study (HCP participants). Interviews lasted approximately 1 h and were audio recorded, transcribed and pseudonymised, so both participant and therapist details were not identifiable without the key file.

Reflexive thematic analysis was conducted in an iterative process. A data‐driven, inductive approach was used initially to capture the experiential aspects and contextual factors, allowing new themes to emerge from the data. A deductive approach was used to collect specific feedback on practical or structural elements. Initial coding was conducted, and themes and subthemes were developed by NZ. The thematic framework was reviewed and revised iteratively with members of the STEADY research team (NZ [mixed‐methods researcher in diabetes and psychology, medical psychology PhD candidate], MS [STEADY chief investigator, clinical academic diabetologist], AH [clinical academic psychologist in eating disorders]) and reviewed and revised further by multidisciplinary researchers and clinicians external to STEADY (GN [clinical academic psychologist specialised in diabetes, mixed‐method expertise], JP [clinical academic psychologist, intervention evaluation expertise] and BdG [clinical academic internist, diabetes expertise]). Researcher reflexivity was an integral part of these discussions, with clinical experiences and interpretations from both diabetologists and psychologists helping to refine codes for both the experiences of participants who received STEADY, as well as the experiences of HCPs and the implementation considerations, from a multi‐country and multidisciplinary perspective. Researchers with theoretical and mixed‐methods expertise helped to shape and structure, refine and reorganise the themes and subthemes. The other authors, mainly clinicians involved in the care of participants and researchers involved in the wider development of STEADY, critically read the ensuing written narrative.

## 3 RESULTS

### 3.1 Participants who took part in the STEADY intervention

#### 3.1.1 Descriptives

Thirteen people (12 women, one man, mean ± SD age 40 ± 11 years; diabetes duration 20 ± 13 years; baseline HbA1c 70.8 ± 27.5 mmol/mol/8.6 ± 2.5%; therapy sessions attended 11.7 ± 0.9) who took part in the STEADY intervention group participated in the process evaluation sub‐study interviews. Eleven participants completed the PROMs. Seven participants received STEADY therapy but did not participate in the process evaluation due to withdrawal from the main trial (*n* = 4) or not consenting to the sub‐study (*n* = 3).

#### 3.1.2 PROM results

The mean (±SD, min–max, range) PROM scores indicate high acceptability (AIM: 18.36 ± 2.01, 14–20), appropriateness (IAM: 18.18 ± 2.64, 12–20) and feasibility (FIM: 18.00 ± 2.32, 14–20).

#### 3.1.3 Semi‐structured interview results

Interview themes were organised into two overarching categories: *Experiences during STEADY therapy*, and *Implementation and structural considerations*.

*Experiences during STEADY therapy* included four main themes (Figure 2; Table 1): (1) Therapeutic growth, (2) Integrated T1DE approach, (3) Therapeutic relationships and (4) Expectations of therapy.

**FIGURE 2:**
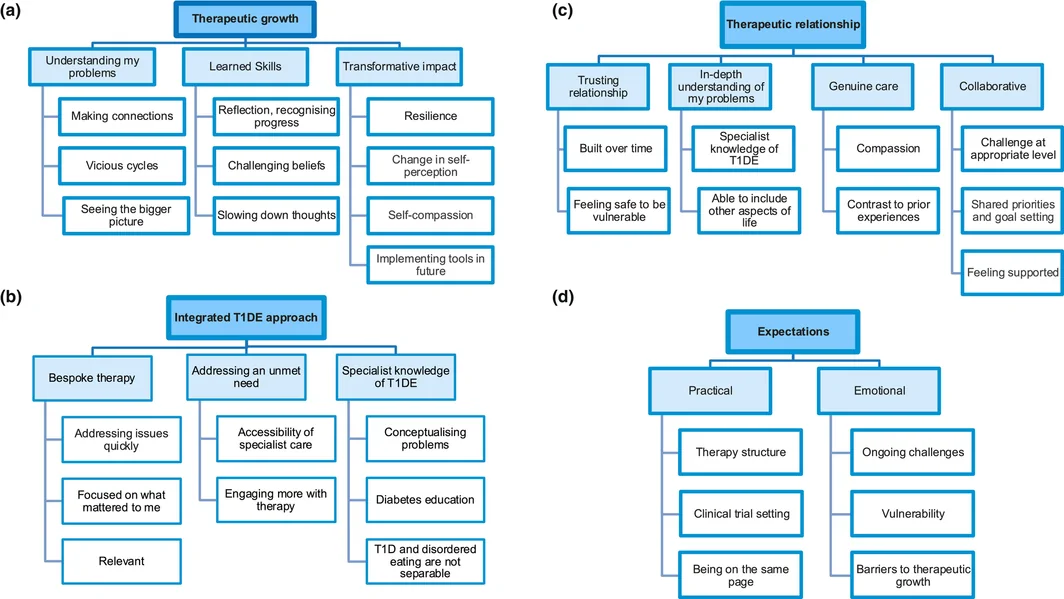
Thematic maps of themes and subthemes in *Experiences in STEADY therapy* for participants receiving STEADY therapy. Subthemes: a. Therapeutic growth, b. Integrated T1DE approach, c. Therapeutic relationship, d. Expectations of therapy. T1D, type 1 diabetes; T1DE, type 1 diabetes and disordered eating.

**TABLE 1:** Themes, subthemes and quotes for ‘Experiences receiving STEADY therapy’ from participants' perspective.

(a) Therapeutic growth—subthemes and quotes	
Understanding my problems	‘Hearing you're a bad diabetic, you're poorly controlled, you're lazy, you're not trying […] I kind of put this label on myself of I'm not enough. […] I didn't realize how many different areas of my life that self belief had encroached into. So it obviously started from my diabetes, but then it went into my academics, my career, my relationships, everything.’ (P6)
Learned skills	‘It was challenging my own thoughts […] not focusing on the numbers so much and having a bit of breathing space. That was really painful in the beginning because I didn't really want to do it, but as time went by, it got easier.’ (P8) ‘… stepping back and really considering the dosages I'm taking and the hypo treatments that I'm taking, that has been a really nice skill, being a bit more conscious of my diabetes management, because I think I was always very quick to just treat and not deal’ (P3)
Transformative impact	‘the issues I have with food are still there, but I am more balanced, I'm more resilient to them and I don't get so overwhelmed by them […] And in terms of my diabetes, it is less burdensome. It's always going to be hard work, but it's not quite so exhausting. And I have a bit more space from it now’ (P3) ‘I feel like my mindset's really changed with regards to how I feel about myself and how I feel about having diabetes.’ (P2)

(b) Integrated T1DE approach—subthemes and quotes	
Specialist knowledge of T1DE	‘you're in a little bit of a tornado of everything circling around. It's like you can't just pull out like the body image part because the body image part is actually related to then the food that you're looking at eating and the insulin that you're having. And so you have to put the food part in there and the insulin part in there.’ (P11) ‘I came from the point of view of 100% [time in range] you won't have complications. And for each percent you come down, something's going to go wrong […] And that's not at all the case. So that was a huge revelation to me in terms of understanding the facts, but also from my mental health.’ (P4)
STEADY addressed an unmet need	‘[prior to STEADY] I was being ping ponged between [the diabetes and eating disorder teams] and not getting any help … [I was referred to a T1DE service] but then they told me that because I hadn't been hospitalized, I wasn't [eligible].’ (P1) ‘I had some CBT before and had reached a real brick wall with it because I hadn't found a therapist who understood diabetes enough. […] I wasn't making more progress because diabetes had so much to do with my disordered eating and my mental health’(P3)
Tailored and relevant	‘with my disordered eating, It's not around anything to do with body image. So as soon as [my therapist] realized that, [she knew] we haven't got to talk about or do because it's not an issue for you. So there was nothing that was irrelevant or I was sat there thinking, this doesn't apply to me.’ (P6) ‘it felt very personal. It felt very bespoke. It was [my therapist] saying at the end of this session, okay, this is why I think you would benefit from filling this [worksheet] out. It felt like she's got thousands of them, and she's picking out specific ones for me, which I thought was very good.’ (P4)

(c) Therapeutic relationship—subthemes and quotes	
Trusting relationship	‘She's very good at listening and making you feel quite normal […] she understands.’ (P5) ‘I don't really trust people, so I just probably didn't say very much to begin with, but at the same time it was nice […] I didn't have to sit there and explain, this is what happens when I have a low […] I didn't have an excuse not to talk about the other stuff that was actually on my mind’ (P8) ‘I find talking about my diabetes really difficult […] I didn't feel at ease with [my therapist]. That never really gelled […] But I also recognize that she's asking me really challenging questions, and so, I think maybe that would have been the same with anybody.’ (P10)
In‐depth understanding of my problems	‘She could see me for who I was, or, am. She could piece together what had happened in my life just to make me what I am now. So it's fascinating that, you know, it wasn't just me overreacting or me being odd or anything.’ (P5) ‘there was a lot of, you know, getting to know you time, but within that, as I said, it was interesting being able to talk to someone who knew what I was talking about, and that was so beneficial.’ (P1)
Collaborative	‘She was challenging the right level, − She didn't impose herself in any way whatsoever. She would chat stuff through, and then she would say, hang on, let's stop. Let's talk about what you said there. Why did you say that? What do you mean by that?’ (P4) ‘It was a real team effort and I didn't have kind of a responsibility to do anything if it wasn't actually working for me. That was a definite benefit and kind of meant that I only ever had a positive association with the sessions’ (P3)
Genuine care	‘one of my biggest takeaways from the study is there are people out there that actually care and want to make a difference’ (P13) ‘I felt [the sessions] were really engaging. Getting to ask how things have been over the week, and not just focused on, oh, how are you getting on with taking insulin? […] a genuine kind of compassion and that was nice because I think you need that in order to be able to share how tough things are.’ (P11)

(d) Expectations of therapy—subthemes and quotes	
Practical expectations	‘in my head, I was like, this is a lapse, this isn't a relapse. This is just a little blip, a little bump in the road […] that could have been my way of thinking, it may not have been quite how everyone else saw it’ (P11) ‘I was very much aware during it that this was for a study. being aware of that maybe made me, sit back a little bit and be like, letting [my therapist] lead it and sort of waiting for her kind of to tell me what to do sort of thing.’ (P9)
Emotional expectations	‘it sounds obvious, but almost more of a disclaimer about how challenging it might be for the participants. I knew it would be hard work, but I don't think I'd quite grasp how hard work.’ (P10) ‘I was sceptical of, but not of the program, but of myself. I think when you've had an eating disorder for a very long time, you just go, like, nothing will help. […] That's a sort of defeatist mentality’ (P1)

#### 3.1.4 Therapeutic growth

Participants had a clear narrative of their path through therapeutic growth (Figure 2a; Table 1a). Participants gained understanding of their problems, where they felt ‘stuck’ and learned about ‘vicious cycles’ throughout therapy. They developed new skills to address their problems and T1DE behaviours (e.g. by challenging unhelpful beliefs). Over time, participants experienced transformative impacts, giving examples of improvements in self‐perception, self‐compassion and resilience. Particularly with diabetes self‐management, participants expressed more confidence and ability to deal with blood glucose fluctuations more effectively, as they received important diabetes education in addition to emotional skills.

#### 3.1.5 Integrated T1DE approach

The integration of T1D and eating disorder specialisms facilitated the therapeutic growth described above, addressing an unmet need in routine services (Figure 2b; Table 1b). CBT‐trained DSN‐delivered therapy allowed participants to use diabetes data therapeutically (e.g. reviewing continuous glucose monitor [CGM] data, making connections with T1DE symptoms), supported diabetes management decisions and provided diabetes education in areas like using diabetes metrics to have a more realistic versus perfectionistic understanding of diabetes management. Some participants described addressing their issues more quickly than in previous therapies due to their therapist's specialist knowledge of T1DE and focus on aspects of therapy that were most meaningful and relevant to them.

#### 3.1.6 Therapeutic relationship

Within the therapeutic relationship, establishing trust was a priority for participants to feel comfortable in therapy, which took time to develop (Figure 2c; Table 1c). For most participants, their therapists' in‐depth understanding of T1DE's diverse presentations and physical and emotional impacts meant that participants felt understood. If a participant felt that trust had been broken or strained with their therapist, it deeply impacted whether they felt safe sharing their experiences or problems and took time to repair. The collaborative approach helped participants feel supported throughout the challenges of therapy and made gradual changes they believed were sustainable. Participants felt their therapist genuinely cared about them, wanted to understand their problems and help and support them in their recovery.

#### 3.1.7 Expectations of therapy

While some participants were familiar with the structure of CBT and welcomed flexibility, others felt less prepared and preferred more explicit rules and pre‐defined structure (Figure 2d; Table 1d). Some participants expected therapy to be less intensive in a research study and were surprised at the depth of STEADY and the progress made in 12 sessions. Clear communication with therapists was important, particularly when participants may have agreed routines or behaviours with their usual care teams that did not align with safety protocols within a trial setting. Emotionally, some participants found the vulnerability of therapy challenging and harder work than expected. Personal circumstances could facilitate or create barriers to engagement, and at times, participants felt unsure of therapy or felt the therapist's time could be used for people with more important problems. Many participants continued to face challenges at the end of therapy and wished for ongoing sessions.

The overarching theme, *implementation and structural considerations* (Figure 3; Table 2) included subthemes: (1) Flexible delivery, (2) Toolkit elements, (3) Active practice, (4) The STEADY app and (5) Additions for future content.

**FIGURE 3:**
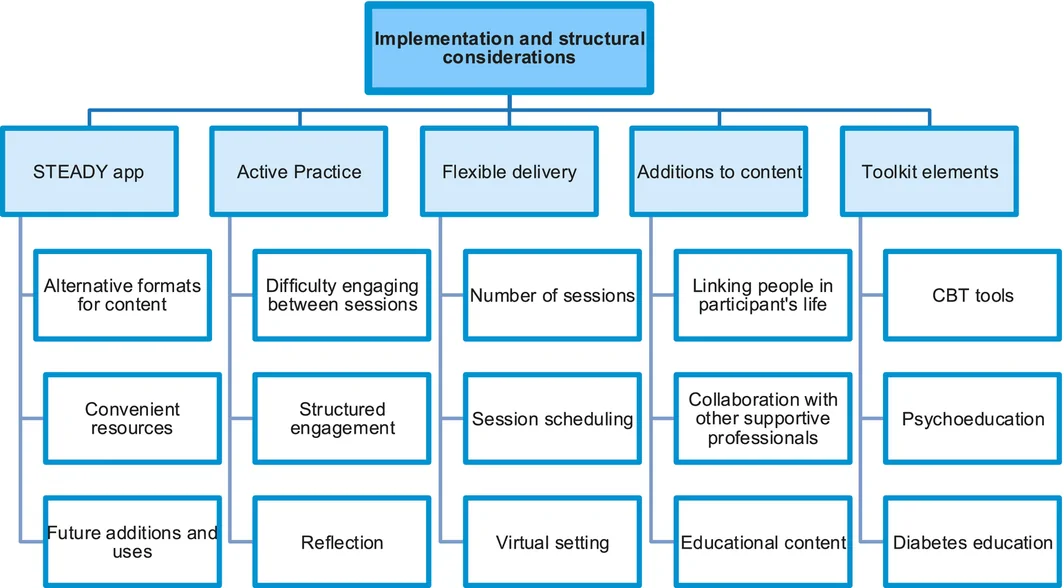
Thematic map of subthemes in *Implementation and structural considerations* for participants receiving STEADY therapy. CBT, cognitive behaviour therapy.

**TABLE 2:** Themes and quotes for ‘Implementation and structural considerations’ from participants' perspectives.

Flexible delivery	‘the twelve weeks definitely was beneficial and helpful, but I think in an ideal world that service would be available lifelong because you go through different challenges and circumstances and changes throughout your whole life and your diabetes isn't going anywhere.’ (P6) ‘There were times where [my therapist] was asking me to think about something and it was more appropriate for me to have two weeks to think about it, or see how those behaviours were happening over time instead of trying to come to a conclusion in a week’ (P8) ‘maybe booking all twelve [sessions] in advance might be helpful.’ (P10)
Toolkit elements	‘I like the use of SMART goals. They didn't have to be these big, out of reach things. It could be little stepping stones, and it feels more manageable than saying, right, you need to drop your Hb4A1c to this by this date.’ ‘It's having that education that wasn't really accessible to me. That has been the biggest thing for me.’ (P2)
Active practice	‘I liked having homework […] I felt like I reflected a lot and I learned a lot. Otherwise, you can forget between one session to another session’ (P12) ‘Part of my life situation as well is that I just finished a degree. I was quite resistant to have homework […] I was struggling to fit in any additional work into my life. And so I just did a lot more reflecting in my own head and, like, talking it through with my friends and family rather than using [the worksheets].’ (P3)
STEADY app	‘It's better than print offs where I've had previous eating disorder sessions […] on your phone, it's there. It's accessible. You've not lost it. It's always in the same place. So, yeah, I think it is good for that reason’ (P13) ‘I think the app could be useful, though, especially if you still had access to it outside of your sessions. If you could still report like a thought diary or something, then if you did need to go back into that kind of service, you could show your practitioner, this is the kind of stuff I've been dealing with.’ (P6)
Additions to future content	‘I think we were talking a bit about hormonal stuff and it might have been nice to have a bit more knowledge of how a woman's menstrual cycle might impact my diabetes because that's something I don't have a very good understanding of’ (P3) ‘It was helpful with [my therapist] to really get under the skin of the problems […] and really important in identifying things, but then having someone else [like my life coach] to talk through the practical solutions was really helpful’ (P10)

#### 3.1.8 Flexible delivery

Participants felt the amount of flexibility in session scheduling was a facilitator to making therapeutic changes, accommodating the demands of their daily lives (e.g. studies, life events) and the therapeutic context of their sessions, allowing participants to feel supported for a longer duration. Virtual delivery helped fit therapy into participants' routines without booking time off work, childcare, or travel. While several participants felt that 12 sessions were sufficient, most felt that more would be needed to sustain change due to the depth of their problems and the lifelong nature of living with T1D.

#### 3.1.9 Toolkit elements

Participants consistently described CBT tools like behaviour experiments, thought diaries and ‘SMART’ goals (Specific, Measurable, Achievable, Relevant, Time‐bound) as important exercises that facilitated change and could be used in and outside of sessions. Strategies to manage difficult emotions, perfectionism, understanding personal values and self‐compassion were key psychoeducational tools. Diabetes education helped participants understand more about diabetes and its impact, including strategies for managing hypoglycaemia and navigating time in range.

#### 3.1.10 Active practice

Conducting active practice between sessions in a structured manner was seen as helpful and engaging for those able to do so; however, some participants found completing exercises between sessions difficult, given the time demands of their daily lives or were unable to complete exercises outside of sessions. Reflection between sessions was common, with some participants using specific prompts, while others used an unstructured approach to reflection or completed structured therapy exercises within sessions.

#### 3.1.11 STEADY app

Most participants downloaded the STEADY app when beginning therapy, but most preferred using other formats for their therapy content, for example, email, writing on paper or in their phone's notes app. The app library was a useful function to have STEADY content in one place and to have a dedicated space to write thought diaries. Suggestions for improving the app included customisable features and notifications, developing the app as a tool for long‐term support and optional linkage with usual care teams.

Lastly, *Additions to future content* included suggestions such as collaboration with other HCPs or supportive professionals, linking with people in participants' daily life, such as flatmates or partners for additional support, and adding additional educational content, for example, managing T1D during the menstrual cycle and the dawn phenomenon.

### 3.2 HCPs involved in the delivery of therapy and psychological assessment

All eligible HCPs took part in semi‐structured interviews and completed the PROMs.

#### 3.2.1 PROM results

Mean (±SD, min–max range) PROM scores indicate high acceptability (AIM: 17.67 ± 0.2.08. 16–20), appropriateness (IAM: 17.33 ± 2.31, 16–20) and feasibility (FIM: 17.67 ± 2.08, 16–20).

#### 3.2.2 Semi‐structured interview results

Interview themes were organised into two overarching categories: *Experiences delivering STEADY therapy*, and *Implementation and structural considerations*.

*Experiences delivering STEADY therapy* had four main themes: (1) Bespoke therapy, (2) Participant's engagement with therapy, (3) Therapeutic skills and (4) Organisational factors (Figure 4; Table 3).

**TABLE 3:** Themes, subthemes and quotes for ‘Experiences delivering STEADY therapy’ from HCPs' perspective.

(a) Bespoke therapy—subthemes and quotes	
Toolkit versatility	‘In the formulation you capture major thoughts and beliefs which it helps orientate people to looking at the way they think, how it relates to their behaviour and these rules they have created’ (HCP1) ‘It was specific enough to the condition and had the advantage of being able to be more personalised person to person. The nice thing about it is that it's an intervention that can potentially be done more than once with different aspects being highlighted’ (HCP2)
Flexibility of delivery	‘everybody's different, everybody's an individual and you see you can use them more as a guidance for the therapist, but you might not share the tools with the person because they just do not, you know that's not the way they roll.’ (HCP1) ‘[the behaviour experiment] template and what you're trying to do is give people skills or help them to learn skills that are going to be useful ongoing. So if you get stuck in the future […] you might be able to use the template and set up your own experiment’ (HCP1)
Qualitative focus	‘[diabetes is] just numbers, numbers, numbers. […] when your whole life has been driven by [HbA1c and time in range] and you never feel that you can achieve them, and then you go to therapy and you get more of the same, it's like, well, this is more of the same.’ (HCP1) ‘if you think about [questionnaires and scoring systems] we want to show change [in their moods …] but when you're navigating the actual therapy, sometimes you need to be a little bit sensitive to people's thinking styles and what perpetuates their problems.’ (HCP1)

(b) Participants' engagement with therapy—subthemes and quotes	
Therapeutic alliance	‘there was sense of the actual process of meeting with clinicians who were aware of their difficulties and having a compassionate understanding for it. That in itself was an important part of the process’ (HCP2) ‘When someone's full of shame [about T1DE], they may find it very hard to speak to you until they feel safe… and then suddenly they'll open up… And it might be session five or six’ (HCP1)
Complexity of presentation	‘So some people I felt I didn't get very far with it all because the complexity of their experience and the emotional content of their experience […] There's an understanding of their situation, but there wasn't perhaps a treatment put into place that would be effective to deal with the severity of their presentation. It would be that it needed more time.’ (HCP1) ‘I went through the perfectionist thing with [a participant] and I thought, wow [they are doing well with this], and then the next session [the participant] said, I can't remember a thing we did because my glucose was really quite high at the time’ (HCP1)
Personal circumstances	‘It was like some catastrophe or some kind of crisis or chaos or something […] it must be very challenging to have to get into the details of carbs and insulin and everything like that when you've got all of that going on. So I did really feel like people were sort of up against the brick wall sometimes.’ (HCP3)

(c) Therapeutic skills—subthemes and quotes	
Specialist knowledge of T1DE	‘a lot of people do not associate their altered eating patterns with their blood sugar levels at all […] so when you've got the [thought] diary which shows the thoughts and the emotions and the behaviour […] then you can look at the [sensor data] and [say] “Can we look at this together with your data and see if we can see anything that's going on here?” and you might see patterns and the two together then become very strong.’ (HCP1)
CBT skills	‘The full formulation, early experiences, diagnosis, is really important to capture, because experiences of diagnosis can be so traumatic […] quite often, people have never been asked the question of the experience of the diagnosis of diabetes’ (HCP1) ‘the function of [T1DE] this is to give you some control […] what's happens as a consequence of that? What's your experience because of that? You know you've put it in place because it makes you feel safe… but actually does it make you feel safe? … then you can start to use behaviour experiments to test other things out. You know, try a different thing.’ (HCP1)
Therapist flexibility	‘You might start a session with a toolkit exercise about weight gain… then you might have to deviate off a bit because the person is putting their own experience on the agenda. And you need to be able to roll with that.’ (HCP1) ‘it was directive. You were actively working on something together, and I think that that made it more collaborative’ (HCP3)

(d) Organisational factors—subthemes and quotes	
Constraints of research trial	‘I think the people having to wait a long time for an intervention was something, you know, obviously not intended, but as we were doing them in blocks and they had to wait for the therapist availability, so I think that had an impact’ (HCP2) ‘for something that's quite complex it's quite [a] low [number of sessions …] And I think this is much more complicated to unravel and to get people to be brave enough to make very big, very fundamental changes […] That's a big ask, really. I think you probably do need a few more sessions’ (HCP3)
Multidisciplinary support	‘we come from very different backgrounds […] so it is playing to those strengths, isn't it? And that's where the shared supervision is so useful […] this is the whole point you're trying to provide the care that they need at one point, rather than saying, well, this is outside of my remit and I can't help you.’ (HCP1)

#### 3.2.3 Bespoke therapy

The therapeutic tools were highly versatile, engaging and adaptable to participants' specific presentations and therapy needs, particularly core CBT tools (e.g. behaviour experiments, formulation), emotional regulation techniques and diabetes education (Figure 4a; Table 3a). Behaviour experiments offered a non‐prescriptive approach that gave participants a template for future use in daily life. A focus on meaningful, qualitative goals was found to be more acceptable than a numbered rating scale or strict numerical goals, as it allowed for more flexible thinking and reduced perfectionist or all‐or‐nothing thinking.

**FIGURE 4:**
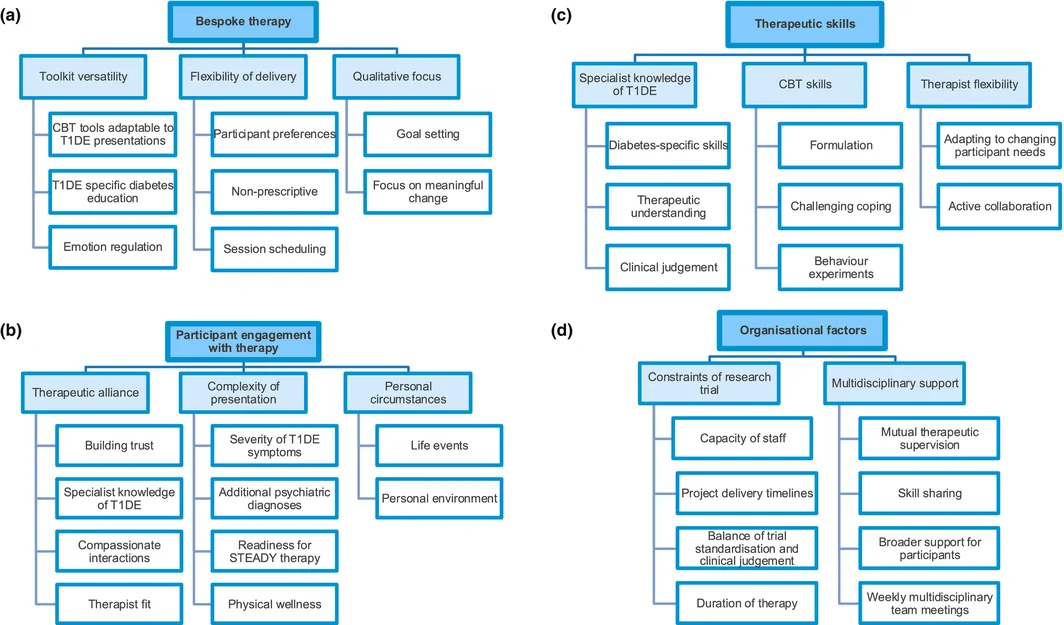
Thematic maps of themes and subthemes in *Experiences delivering STEADY therapy* for healthcare professionals delivering therapy. Subthemes: a. Bespoke therapy, b. Participant engagement with therapy, c. Therapeutic skills, d. Organisational factors. CBT, cognitive behaviour therapy; T1DE, type 1 diabetes and disordered eating.

#### 3.2.4 Participant engagement with therapy

The therapeutic alliance was an important relational factor in how HCPs understood participants' engagement in therapy (Figure 4b; Table 3b). Building trust over time required consistently proving to participants that they understood the complexity of their unique problems and presentation of T1DE, had the specialist skills and knowledge to address them and compassion for their experiences. Therapist fit was important, with each therapist having distinct experiences and clinical skills to support participants with a specific need. Factors such as comorbidity, traumatic events or ambivalence to therapy meant therapists spent more time preparing participants for therapy and developing the therapeutic formulation, with fewer sessions available for active therapy. Physical wellness impacted engagement and the therapeutic agenda when a participant was unwell or had high glucose during sessions, prioritising managing ketones or participant safety over active therapy. Personal environment, such as workplace flexibility or caring responsibilities, could facilitate or create barriers to engagement with therapy, in addition to life events and disruptions to usual routines and self‐care.

#### 3.2.5 Therapeutic skills

Specialist knowledge of T1DE, in particular diabetes‐specific skills (e.g. understanding sensor data, how diabetes impacts the body, managing insulin changes), informed the therapist's understanding of participants' experiences and guided their approach to therapy (Figure 4c; Table 3c). Clinical judgement was a key requirement for managing medical risk, as well as guiding appropriate changes in diabetes management. Similarly, therapist flexibility was required to be able to manage changing participant needs over the course of therapy or to set shared and sustainable priorities. Cognitive‐behavioural principles were foundational skills therapists used to guide participants throughout therapy, understand the function of their behaviours and build participants' skills in challenging unhelpful thinking and behaviours and building a more compassionate view of self.

#### 3.2.6 Organisational factors

Delivering bespoke STEADY therapy within a research trial presented challenges with randomisation timelines and funding deadlines influencing the pacing of therapy and staff capacity within project timelines (Figure 4d; Table 3d). The clinical judgement and flexibility required to deliver a complex intervention in a cohort with a wide range of T1DE presentations is in opposition to typically rigid and standardised RCT protocols, making standardisation between participants difficult. Twelve sessions are considered a standard course for CBT, but are likely insufficient for people with more clinical complexity in T1DE. The range of therapeutic skills within the multidisciplinary STEADY team was valuable, with the DSN and clinical psychologist able to assess participant needs and consider which therapist was best placed to deliver therapy, and access additional support through weekly meetings with the wider study team. The therapists were able to share skills and provide mutual clinical supervision for the participants under their care, which led to therapists feeling supported and further developing their skills and understanding of T1DE.

The overarching theme, *implementation and structural considerations* had four main themes: (1) Therapist support, (2) Adaptations from standard CBT therapy, (3) Training and HCP needs for wider implementation and (4) Additions and changes to toolkit (Figure 5; Table 4).

**TABLE 4:** Themes and quotes for ‘Implementation and structural considerations’ from HCPs' perspectives.

Therapist support	‘[for some participants] there was more needed beyond our scope of the intervention, and not that the intervention couldn't still happen, but that there needs to be something else kind of providing additional support in the community […] You really need to be mindful of risk if you are providing a remote intervention that is on screen and someone could just close their laptop and don't know what's happening behind that.’ (HCP2) ‘this back‐to‐back seeing patients, we do it in diabetes clinic. You just can't do it with [these participants …] you've got to be able to think things through and frame things properly and maintain that credibility with them.’ (HCP1)
Adaptations from standard CBT	‘I think the online platform is very positive in lots of ways because it's very flexible to patients needs and they don't have to be coming up to the hospital […] not travel[ing] means that they're more likely to attend their therapy […] And I feel that probably meeting them face to face once would be a good thing you know and as an initial assessment, I think you get a better picture of who they are.’ (HCP1) ‘those with very high HbA1c you can't change things quickly because of the risk of complications, exacerbation of complications […] So you've got to do really small steps’ (HCP1) ‘sometimes you'd have to have a session to actually just sort of encourage a participant to put a pump back on or you know test ketones, so you couldn't really use like the CBT study tools as such’ (HCP3)
Training and HCP needs for wider implementation	‘[in existing services] there's a lot of fear around the risk that might be involved with [T1DE …] there certainly needs to be a certain amount of time allocated for training and ongoing support supervision […] all the usual barriers to extending services and developing services will come into play. But, a lot of fear comes from lack of knowledge, information.’ (HCP2) ‘you need a reasonable working knowledge of diabetes. […] if you don't have that, I think that will be very difficult, because I don't think you'd understand the mechanisms like hyper‐ and hypoglycaemia and what it feels like for them, what it does, their weight and shape.’ (HCP3)
Additions and changes to toolkit	‘you can perhaps within therapy think of goals in a slightly different way, “I want to be able to stay awake a little bit longer in the evening so I can do the homework with my child” for example […] rather than “I spend 2 h with my child every evening and that my blood sugar is this number when I do it”… it may be harder to define change, but it's very meaningful change to the person and that's the important part.’ (HCP1) ‘you've got to really work out, how am I going to help this person deal with these emotions that they've been protected from [with their T1DE], and keep them on track, which I think is a major point that we can develop and make more robust in the toolkit.’ (HCP1)

#### 3.2.7 Therapist support

Collaborating with participants' usual care teams to share progress and discuss helpful strategies offers an opportunity to support therapists and participants between sessions, after therapy end or times where more clinical contact is important to manage Diabetic Ketoacidosis (DKA) or mental health risk (Figure 5a; Table 4). The STEADY multidisciplinary team has a role in ensuring there is adequate time and breadth of skills required for supervision, handover of participants from baseline to commencement of therapy and maintaining the capacity of therapy and supervisory staff. Assessment of participants at baseline is essential to ensure that T1DE‐specific CBT is an appropriate course of treatment, or whether the participant requires any other support or preparatory work prior to beginning active therapy. Additional support to reduce the administrative load on therapists would be helpful in the future to increase capacity for therapy delivery.

**FIGURE 5:**
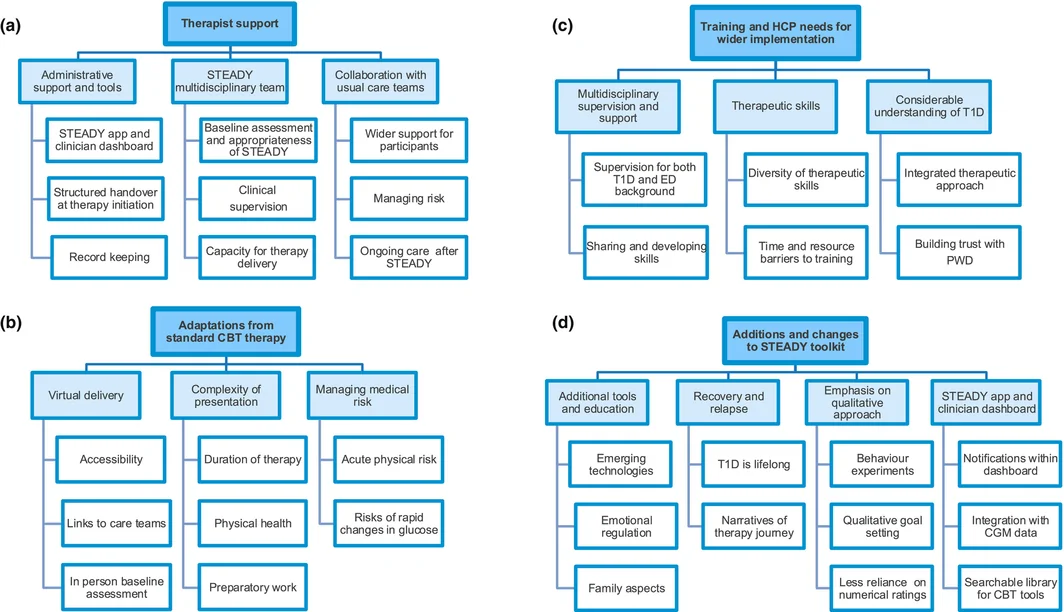
Thematic maps of themes and subthemes in *Implementation and structural considerations* for healthcare professionals delivering therapy. Subthemes: a. Therapist support, b. Adaptations from standard CBT therapy, c. Training and HCP needs for wider implementation, d. Additions and changes to STEADY toolkit. CBT, cognitive behaviour therapy; CGM, continuous glucose monitor; ED, eating disorder; HCP, healthcare professionals; PWD, people with diabetes; T1D, type 1 diabetes.

#### 3.2.8 Adaptations from standard CBT therapy

The virtual delivery improved accessibility and allowed both therapists and participants to be flexible (Figure 5b; Table 4). To support virtual delivery, HCPs recommended closer communication with usual care teams to help manage acute risk, and maintaining in‐person baseline assessments with future participants. The complexity of T1DE presentations required flexible changes to the standard CBT approach. As participants' physical health at times required deviation from the standard CBT agenda, the 12‐session duration of therapy requires clinical discretion for participants who have more complex therapeutic needs. This could include preparatory work prior to active therapy, for example, to manage hyperglycaemia prior to sessions, engage the participant in feeling ready to make changes or seek treatment for additional comorbidities. Changes to diabetes care for people with elevated glucose levels must be done gradually to minimise the risk of complications, requiring a longer duration of therapy.

#### 3.2.9 Training and HCP needs for wider implementation

Multidisciplinary team working was essential for therapists to adequately support participants, sharing skills and knowledge as well as clinical supervision, and would be a key consideration for delivering STEADY in the context of existing services (Figure 5c; Table 4). Given that participants may have complex mental or physical health comorbidities in addition to T1DE, a breadth of skills is necessary for appropriate management, and therapists suggested skills in therapeutic approaches beyond CBT may also be useful. HCPs acknowledged that this training is resource‐intensive, which may present barriers to wider implementation. In addition, HCPs delivering therapy require considerable knowledge of T1D and its physical and psychological impacts. HCPs noted that participants appreciated not needing to explain diabetes or its treatment and that a surface‐level knowledge was not sufficient to provide this type of intensive therapeutic support.

#### 3.2.10 Additions and changes to the toolkit

A meaningful, non‐quantitative approach was emphasised, with a focus on behaviour experiments and curiosity over pre‐defined or prescriptive worksheets (Figure 5d; Table 4). Sessions around recovery and relapse management require distinction from standard CBT, as T1D is a lifelong condition and will present challenges beyond recovery from T1DE symptoms or behaviours. The STEADY app and clinician dashboard have the potential to be useful tools for therapists and participants, but require some adaptation, including notifications when sending messages, customisable features and integration with CGM data. The clinician dashboard also has the potential to be an efficient searchable library for therapy tools and education to improve ease of use during therapy sessions. HCPs recommended additional tools for development in future iterations of the toolkit, with topics including technology and emerging treatments, further resources on emotional regulation and embodiment of symptoms, general psychoeducation and family aspects.

## 4 DISCUSSION

STEADY participants and therapists reported overall positive experiences with the intervention, with high quantitative ratings of the acceptability, appropriateness and feasibility of the therapy and in their qualitative reports.

Participants' and HCPs' experiences were largely complementary, with similar understandings of how STEADY worked and key aspects important in its delivery. Key contextual factors influencing delivery included participants' personal circumstances, complexity of T1DE presentation and a need for a longer duration of therapy for some participants. Key mechanisms of intervention reported were the integrated T1DE approach and highly flexible, personalised CBT delivery, particularly specialist knowledge of T1DE and strong therapeutic alliance. Implementation was facilitated through flexible approaches tailored to participants' needs, multidisciplinary training and clinical supervision, with clinical discretion to deviate from standard CBT practices and timelines to support participants' needs and reduce the risk of diabetes complications. The considerable overlap in themes between participants and HCPs suggests a shared understanding of the intervention processes and impacts, reflecting high intervention fidelity and a model that resonates with both groups. Both gave specific feedback for future iterations of STEADY and implementation. Our findings align with the MRC's framework of context, mechanisms and implementation, and highlight the importance of evaluating interventions at the feasibility stage.^11^, ^13^ The consistency between perspectives in our study offers a strong message for future revisions of STEADY and strengthening delivery.

Our results contextualise the findings of the quantitative audit and evaluation of STEADY delivery.^14^ The CBT formulation, behaviour experiments and thought diaries were consistently referred to by STEADY participants and therapists in our interviews and were the most frequently recorded tools by therapists in the quantitative audit.^14^ Similarly, we also found that managing physical health risks may take priority over standard CBT practices, leading to changes from the set CBT agenda and standard practice in session, or changes to wider therapy structure, such as breaks from active therapy.^14^

Key recommendations for wider clinical consideration emerged in this study. Multidisciplinary clinical supervision was a recurring factor to support therapists in managing the variety of complex presentations in STEADY. The DSN and clinical psychologist delivering therapy benefited from clinical supervisory support and were able to share skills and approach a wider range of T1DE‐specific problems. This is important for future practice to build in specific multidisciplinary supervision when treating people with T1DE and other complex comorbidities. Therapist fit is also a key consideration, as therapists' professional experiences were different and complementary in the skills they presented and the types of issues they felt best able to support. While STEADY was not powered to compare DSN‐ and clinical psychologist‐delivered therapy, future trials should explore how therapists with different backgrounds can optimally support people with T1DE alongside multidisciplinary supervision. Considering participant readiness and engaging in preparatory work before therapy can help individuals feel supported and begin CBT at an appropriate time. This may involve STEADY clinicians working to build trust, or usual care teams to better understand and support participants outside therapy. Preparatory work might also include adjustments to diabetes management or other therapies prior to STEADY CBT, and is a key area where usual care teams may be able to support recovery and readiness for future therapy.

HCPs acknowledged that the specialist T1DE training and additional resources may be difficult for teams to access, as most usual care settings do not have the level of specialist staff and capacity to deliver such intensive and flexible therapy. However, this process evaluation also highlights opportunities for HCPs to adapt existing practices to support people with T1DE in routine care. Multidisciplinary team working and effective communication between services, building trusting relationships with patients over time, making small sustainable changes and focusing on meaningful change are transferable approaches that may be useful for healthcare teams working with people with T1DE, diabetes distress or other challenges with diabetes self‐care, and other complex conditions. As these key factors have been highlighted by both people with T1DE as well as HCPs, they are important and highly implementable opportunities to improve care and highlight the importance of person‐centred approaches.

Participants emphasised the importance and novelty of the integrated T1DE approach to therapy. In previous studies, people with T1DE often reported falling between two services without integration and therefore received disjointed and conflicting care.^4^ Integrated care also impacted the therapeutic relationship, reflecting literature within the wider population of people receiving therapeutic interventions for eating disorders. While there is mixed evidence on the impact of the therapeutic alliance in different eating disorder populations,^16^, ^17^ our research highlights factors that may influence the development of the therapeutic relationship in people with T1DE. Given the importance of T1DE integration and specialist knowledge in building trust between participants and therapists in our study, it is likely these factors influence the agreement of goals, tasks and quality of bond between therapist and individual, defining the therapeutic alliance.^18^ These factors should be explored in future research and may apply to other interventions requiring specialised knowledge of T1D and other comorbidities.

A key strength of this study is the multidisciplinary team involved in the data analysis and interpretation. The varied clinical and research backgrounds helped to safeguard rigour in the analysis and interpretation of these results. Participant themes were also reviewed by the STEADY patient and public involvement and engagement (PPIE) group members and STEADY co‐design participants. Methodologically, the mixed‐method approach and inclusion of validated PROMs enable comparison to other novel interventions and future iterations of STEADY. Including perspectives from both participants and HCPs provided a holistic understanding of how the intervention works in practice.

A key limitation is that we were unable to recruit people with T1DE who were eligible for STEADY but declined participation in the RCT, as planned in the original protocol.^9^ As a result, we do not have data on perceptions of STEADY from people who did not wish to take part. Based on PPIE group discussions and emails during the recruitment phase of STEADY, reasons individuals declined may be (1) not willing to be randomised, (2) past negative experiences with DSNs or diabetes specialists and (3) not ready to engage with therapy. Gaining insight from these individuals in the future could help refine STEADY and better meet the needs of people with T1DE.

The findings of this mixed‐methods process evaluation suggest that STEADY is acceptable, appropriate and feasible to be delivered. Our findings inform the context and mechanisms of therapeutic growth, and implementation considerations that support the further development, testing and future evaluation of the STEADY intervention in a large‐scale RCT.

## FUNDING INFORMATION

This work was conducted as part of the National Institute for Health Research (NIHR) funded STEADY project (Safe management of people with Type 1 diabetes and EAting Disorders studY; CS‐2017‐17‐023 awarded to MS). This process evaluation sub‐study was supported by a seed grant fund (SG‐310123‐LG‐AF‐Zaremba) awarded to NZ by King's Health Partner's Diabetes Endocrinology and Obesity Seed Grant Funding scheme. NZ's salary was in part funded by NIHR (CS‐2017‐17‐023); JT and KI are in part funded by the NIHR Mental Health Biomedical Research Centre at South London and Maudsley NHS Foundation Trust and King's College London. AH's and JB's salaries were in part funded by the NIHR Clinician Scientist Award (CS‐2017‐17‐023) to MS. The views expressed are those of the author(s) and not necessarily those of the NHS, the NIHR or the Department of Health. AH is supported by the Medical Research Council.

## CONFLICT OF INTEREST STATEMENT

None to declare.

## Data Availability

The data that support the findings of this study are available on request from the corresponding author. The data are not publicly available due to privacy or ethical restrictions.
